# Progranulin suppresses titanium particle induced inflammatory osteolysis by targeting TNFα signaling

**DOI:** 10.1038/srep20909

**Published:** 2016-02-11

**Authors:** Yun-peng Zhao, Jian-lu Wei, Qing-yun Tian, Alexander Tianxing Liu, Young-Su Yi, Thomas A. Einhorn, Chuan-ju Liu

**Affiliations:** 1 Department of Orthopaedic Surgery, New York University Medical Center, New York, NY, 10003, USA; 2 Department of Orthopaedic Surgery, Qilu Hospital, Shandong University, Jinan, Shandong, China; 3 Department of Orthopaedic Surgery, Medical School of Shandong University, Jinan, Shandong, China; 4 Department of Cell Biology, New York University School of Medicine, New York, NY 10016, USA

## Abstract

Aseptic loosening is a major complication of prosthetic joint surgery, characterized by chronic inflammation, pain, and osteolysis surrounding the bone-implant interface. Progranulin (PGRN) is known to have anti-inflammatory action by binding to Tumor Necrosis Factor (TNF) receptors and antagonizing TNFα. Here we report that titanium particles significantly induced PGRN expression in RAW264.7 cells and also in a mouse air-pouch model of inflammation. PGRN-deficiency enhanced, whereas administration of recombinant PGRN effectively inhibited, titanium particle-induced inflammation in an air pouch model. In addition, PGRN also significantly inhibited titanium particle-induced osteoclastogenesis and calvarial osteolysis *in vitro, ex vivo* and *in vivo*. Mechanistic studies demonstrated that the inhibition of PGRN on titanium particle induced-inflammation is primarily via neutralizing the titanium particle-activated TNFα/NF-κB signaling pathway and this is evidenced by the suppression of particle-induced IκB phosphorylation, NF-κB p65 nuclear translocation, and activity of the NF-κB-specific reporter gene. Collectively, these findings not only demonstrate that PGRN plays an important role in inhibiting titanium particle-induced inflammation, but also provide a potential therapeutic agent for the prevention of wear debris-induced inflammation and osteolysis.

Total joint arthroplasty (TJA) is one of the most widely performed elective surgeries for the treatment of severe joint diseases such as osteoarthritis and rheumatoid arthritis, providing effective pain relief and functional improvement in patients’ daily lives^1^. Annually, approximate 1.5 million hip and knee arthroplasties are performed worldwide, and the demand for these procedures is increasing^2^. Despite several recent advances, revision of TJAs remains a major concern. Aseptic loosening and periprosthetic osteolysis are the leading causes of arthroplasty failure, which occurs as a result of the biological response to particulate wear debris such as titanium particles^3^,^4^. Although the pathogenesis of the ensuing aseptic loosening remains unclear, TNFα is involved in the pathogenesis of the osteolytic response and is believed to play an important role in the inflammatory osteolytic process^5^. TNFα is dramatically induced by the presence of wear debris particles, and is detectable in the fluid and inflamed tissues surrounding loosened implants^6^. It is well established that TNFα signaling is crucial to the wear debris-induced inflammatory osteolytic response and considerable evidence suggests that periprosthetic osteolysis iss initiated by the activation of the nuclear factor kappa-light-chain-enhancer of activated B cells (NF-κB) signaling pathway^7^,^8^. Moreover, anti-TNFα agents have been tested in wear debris-induced osteolysis models and exhibited effective inhibition of osteolysis^9^.

Progranulin(PGRN), also known as granulin epithelin precursor (GEP), PC-cell-derived growth factor (PCDGF), proepithelin, and acrogranin, is a 593-amino-acid autocrine growth factor^10^. PGRN affects TNFα-mediated signaling pathways by inhibiting the binding of TNFα to TNFR1/2 and exhibits anti-inflammatory function in various kinds of diseases and conditions^11^,^12^,^13^, including inflammatory arthritis models^14^. Furthermore, the administration of recombinant PGRN exhibits anti-inflammatory effects in various inflammatory diseases^14^,^15^,^16^,^17^,^18^,^19^,^20^,^21^,^22^

Given the importance of the TNFα/NF-κB signaling pathway in the pathogenesis of inflammatory osteolysis and PGRN’s anti-TNF activity, we hypothesized that PGRN might represent a novel treatment for titanium-induced inflammatory osteolysis. In this study, we examined the expression pattern of PGRN following induction of titanium particle-induced inflammation both *in vitro* and *in vivo*, and determined the effects of endogenous and exogenous PGRN in titanium-induced inflammatory osteolysis as well as the signaling pathways involved.

## Methods and Materials

### Media, reagents, and cells

Dulbecco’s Modified Eagle Medium (DMEM) (SKU# 11965–118) and fetal bovine serum (FBS) (catalog# 16000–044) were purchased from Gibco-BRL (Waltham, USA). Specific antibodies against progranulin (PGRN) (catalog# sc-28928), COX-2(catalog# 514489), NOS-2(catalog# sc-649), p-IκB(catalog# 4094), and glyceraldehyde-3-phosphate dehydrogenase (GAPDH)(catalog# 25778) were obtained from Santa Cruz (California, CA, USA). β-Tubulin(catalog# 2146) was obtained from Cell Signaling Technology(Danvers, USA). ELISA kits of IL-6(catalog# 88–7046), TNF-α(catalog# 88–7324) and IL-1β(catalog# 88-7013) were obtained from eBioscience (San Diego, CA, USA). Tris(catalog# T1503), glycine(catalog# G8898), sodium dodecyl sulfate (SDS) (catalog# T7777), tartrate-resistant acid phosphatase (TRAP) staining kit(catalog# 387A-1KT) , and other regents were obtained from Sigma (St. Louis, MO, USA) unless stated otherwise. RAW264.7 cells (catalog# 85062803-1VL) were purchased from Sigma (St. Louis, MO, USA).

### Particle preparation

**Figure 1:**
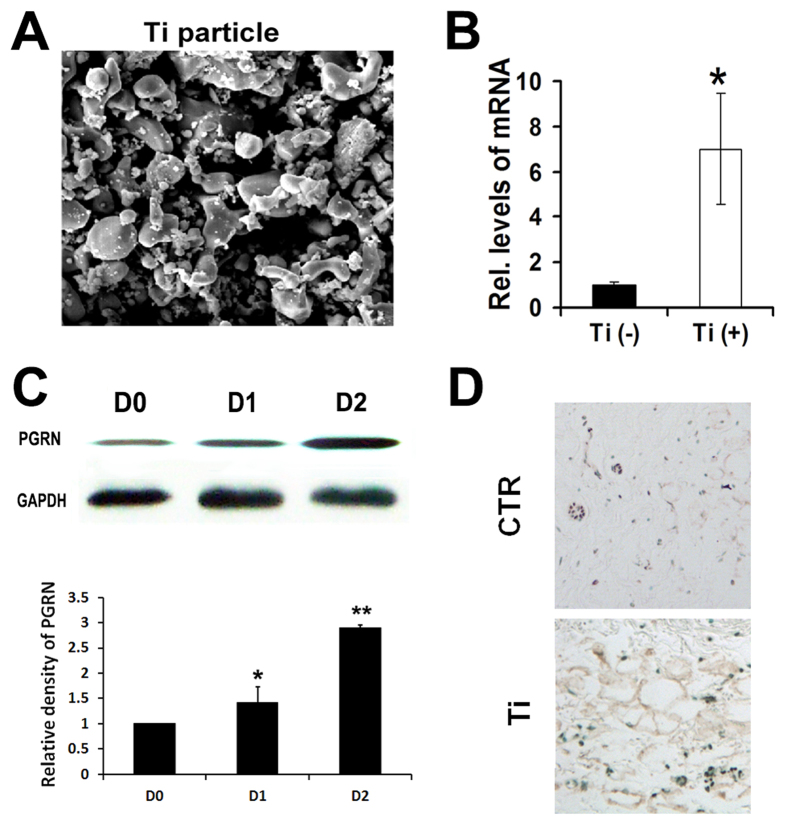
Titanium particles up-regulate PGRN both *in vitro* and *in vivo*. (**A**) The scanning election microscopy (SEM) appearance of the titanium particles (magnification × 5000). (**B**) mRNA levels of PGRN in RAW264.7 cells stimulated by Titanium particles, assessed by real-time PCR. (**C**) Protein levels of PGRN in RAW264.7 cells stimulated by Titanium particles, assayed by Western blotting. Relative band density was analyzed using imageJ program. (**D**) Increased expression of PGRN in Titanium stimulated air pouch membrane, assayed by Immunochemistry staining. The values are mean ± SEM of at least 3 independent experiments. *p < 0.05, **p < 0.01.

Pure Ti particles were purchased from Johnson Matthey chemicals (catalog #00681, Ward Hill, Massachusetts, USA; Fig. 1). Ninety percent of the Ti particles were <3.6 μm in diameter, 75% were <2.4 μm, 50% were <1.6 μm, 25% were <1.2 μm, and 10% were <1.0 μm. Ti particles were prepared as previously described, and the particles tested negative for endotoxin using a Limulus Amebocyte Lysate Kit (BioWhittaker, Walkersville, MD) as described previously^23^,^24^. Sterile particles were suspended in phosphate buffered saline (PBS) and stored at 4 ^o^C before use.

### Generation of a PGRN stable line and purification of recombinant PGRN protein

The method to generate a PGRN stable line and purify recombinant PGRN has been described in our previous publication^25^.

### Animals and surgery

All animal studies were performed in accordance with institutional guidelines and approval by the Institutional Animal Care and Use Committee of New York University. The generation and genotyping of PGRN-deficient mice have been described previously^14^,^26^. 8 weeks old mice were used for this experiment.

### Titanium particles stimulated the mouse air pouch model

Titanium particles induced (Ti-induced) air pouches were generated according to the method as described previously^27^. The dorsal skins of wild type and PGRN-deficient mice were cleaned and shaved to provide a donor site. 3 ml sterilized air was injected subcutaneously to form an air pouch at this area. The air pouches were injected with 1 ml air every other day for 5 days. On the second day, 5 mg Ti particles suspended in 0.5 ml PBS were injected into pouches to provoke inflammation. 0.5 ml PBS was injected into the control pouches. For the mice with drug treatment, PGRN (200 μg/kg) was injected into pouches daily until sacrifice of the mice. Each group comprised 10 mice. Mice were sacrificed 7 days after the model was established. The pouch membranes were harvested for molecular and histological analysis.

### Neonatal mouse calvaria osteolysis model

A modification of the technique described previously^28^ was used to examine calvaria cultures. Stimulated medium was prepared by incubation of RAW264.7 cells in the serum-free DMEM medium containing Ti particle (1 × 10^8^ particles/ml) for 3 days. Stimulated medium was then harvested and stored at −80 ^o^C until use. Calvaria were collected from 5-day old postnatal C57BL/6 wild type mice and dissected free of soft tissue. Stimulated medium from activated RAW264.7 cells was diluted 1: 1 in serum-free DMEM and incubated with calvaria for 4 days. To investigate whether PGRN inhibits stimulated medium-induced bone resorption, calvaria were cultured in the medium with or without PGRN (200 ng/ml). Calvaria cultured without PGRN were included as a positive control. In addition, one group of bones was cultured in serum-free DMEM alone for 4 days as negative control. Cultured calvaria were then harvested for TRAP staining or collected for real-time PCR.

### Calvarial osteoysis moue model

The calvarial osteolysis model was established as published previously^29^. Briefly, an area of skin overlying the skull was carefully shaved and sterilized with betadine scrub. For anesthesia, intraperitoneal injections of 15 mg/ml ketamine and 1 mg/ml xylazine were dosed at 10 ml/kg body weight. The surgical area was manually depilated and disinfected. A 0.5 cm sagittal incision was made and the periosteum remained intact. Using sterile technique, a 25-gauge needle was used to inject 100 μl of PBS with or without 20 mg Ti particles resuspended in it directly over the calvarial bone and periosteum. 10 control mice received PBS injections (sham), and 10 control animals received 20 mg Ti particle resuspended in PBS (Ti only). 10 animals received 20 mg titanium particles as well as intraperitoneal injection of PGRN (200 μg/kg) every other day from day 0 (Ti + PGRN). 8 days postoperatively, the mice were euthanized, and the calvaria harvested for micro-CT analysis, protein quantification, and histological analysis.

### *In vitro* osteoclast differentiation

Induction of osteoclastogenesis was performed as previously described^14^. Briefly, RAW 264.7 cells were maintained in Dulbecco’s modified Eagle’s medium (DMEM) (Gibco BRL, MD) containing 10% fetal bovine serum (FBS) at 37 °C in a humidified incubator with 5% CO_2_. To induce osteoclast differentiation, RAW 264.7 cells were cultured in presence of 50 ng/ml receptor activator of nuclear factor kappa-B ligand (RANKL). To investigate whether titanium particles could enhance RANKL-mediated osteoclastogenesis, RAW 264.7 cells were cultured in presence of 50 ng/ml RANKL and 1% titanium particles. To determine whether PGRN could suppress titanium particles-enhanced osteoclastogenesis, RAW 264.7 cells were cultured in presence of 50 ng/ml RANKL and 1% titanium particles and 500 ng/ml PGRN. Cells were harvested and followed by TRAP staining after 7 days culture.

### RAW 264.7 cell culture and stimulation

RAW 264.7 cells were maintained in Dulbecco’s modified Eagle’s medium (DMEM) (Gibco BRL, MD) containing 10% fetal bovine serum (FBS) at 37 °C in a humidified incubator with 5% CO_2_. To investigate the effects of particle stimulatation on an array of mRNA gene transcripts, RAW 264.7 cells were cultured in the absence or presence of 500 ng/ml PGRN with l% Ti particles dissolved in the same medium for 6 h before RNA extraction^14^,^30^,^31^,^32^. Protein was extracted after 24 h and 48 h of Ti particles stimulation. We also used 500 ng/ml etanercept (Enbrel) as a positive control.

### Micro-CT

Prior to histological processing, paraformaldehyde-fixed calvaria from each group were evaluated with micro-CT using a Scanco vivaCT40 cone-beam scanner (SCANCO Medical, Switzerland) with a 55 kVp source and a 145 μAmp current as we described before^25^. We scanned the calvaria at a resolution of 10.5 μm. The scanned images from each group were evaluated at the same thresholds to allow 3-dimensional structural reconstruction of each sample. The osteolysis in calvaria of each treatment group was analyzed through structural reconstruction.

### Histology

The calvaria and air pouch from all experimental groups were fixed in 4% paraformaldehyde, decalcified, dehydrated, cleared with dimethylbenzene, and then embedded in olefin. At least 4 consecutive 6-μm sections were obtained from the sagittal planes, and stained using hematoxylin and eosin (HE), Masson Tricherome and Tartrate Resistant Acid Phosphatase (TRAP) .

### Real-time RT-PCR

Total RNAs were extracted from RAW 264.7 cells or skin or skull tissues using an RNeasy kit (Qiagen, Valencia, CA, USA), and reverse transcription was performed using a RT-for-PCR kit (Qiagen, Valencia, CA) following the manufacturer’s protocol. Reactions were performed in a 20-μl SYBR Green PCR volume in a 96-well optical reaction plate formatted in the 7300 Sequence Detection System (Applied Biosystems, Foster City, CA, USA). The primers for real time PCR used were listed as followings:

PGRN, 5′-TGGTGGAGCAGCAAGAGCAA-3′ and 5′-CAGTGGACAGTAGACGGAGGAAA-3′; IL-1β, 5′-AATCTCACAGCAGCACATCA-3′ and 5′-AAGGTGCTCATGTCCTCATC-3′; IL-6-F, 5′-ATGAAGTTCCTCTCTGCAAGAGACT-3′ and 5′-CACTAGGTTTGCCGAGTAGATCTC-3′; COX-2, 5′-AATGCTGACTATGGCTACAAAA-3′ and 5′-AAAACTGATGCGTGAAGTGCTG -3′; NOS-2, 5′-CAGCCTCTGTCTCTCAGGCTCTT-3′ and 5′-CTCTCTAAGTGAACAACTGGCCTGTGA-3′; NF-KB2, 5′-GCTTCCCGGATTCTCCTAGAC-3′ and 5′-CATACAGGTGTAAGGCAGCAGAGG-3′; TRAP, 5′-CTGGAGTGCACGATGCCAGCGACA-3′ and 5′-TCCGTGCTCGGCGATGGACCAGA-3′; Cathepsin K, 5′-CAGCAGAACGGAGGCATTGA-3′ and 5′-CTTTGCCGTGGCGTTATACATACA-3′; Calcitonin receptor, 5′-CAAGAACCTTAGCTGCCAGAG-3′ and 5′-CAAGCACGCGGACAATGTTG-3′; GAPDH, 5′-ACCCAGAAGACTGTGGATGG-3′ and 5′-CACATTGGGGGTAGGAACAC-3′. The mRNA expression was normalized to GAPDH. The presence of a single specific PCR product was verified by melting curve analysis and for each gene; the experiments were repeated three times.

### Immunohistochemistry

Interface membrane tissue of mouse models were harvested and fixed in 4% PBS buffered paraformaldehyde at 4 °C overnight. After the tissue was dehydrated and embedded in paraffin, 6-μm sections were cut. Thereafter, sections were deparaffinized by xylene immersion, rehydrated by graded ethanol and treated with 0.1% trypsin for 30 minutes at 37 °C. After blocking in 20% goat serum for 60 minutes at room temperature, sections from air pouch model were incubated with anti-PGRN polyclonal antibody^33^ (1:100 dilution; Santa Cruz Biotechnology) and anti-phosphorylated IκB-α (pIκB-α) polyclonal antibody (1:100 dilution; Santa Cruz Biotechnology) at 4 °C overnight, followed by incubation with a horseradish peroxidase–conjugated secondary antibody for 60 minutes at room temperature. The signal was detected using the Vector Elite ABC Kit (Vectastain; Vector).

### Western blotting

Total air pouch membranes and RAW 264.7 cell extracts were homogenized and proteins were collected. Proteins were resolved on a 10% SDS-polyacrylamide gel and electroblotted onto a nitrocellulose membrane. After blocking in 5% nonfat dry milk in Tris buffer-saline-Tween 20 (10 mM Tris-HCl, pH 8.0; 150 mM NaCl; and 0.5% Tween 20), blots were incubated at room temperature with polyclonal anti-PGRN(1:1000 dilution, Santa Cruz Biotechnology), anti-COX-2(1:1000 dilution, Santa Cruz Biotechnology), anti-NOS-2(1:1000 dilution, Santa Cruz Biotechnology), anti-p65(1:000 dilution, cell signaling) anti-GAPDH (1:000 dilution, Santa Cruz Biotechnology)or anti-β-tubulin (1:000 dilution, Santa Cruz Biotechnology) for 1 h. After washing, the secondary antibody (horseradish peroxidaseconjugated anti-rabbit immunoglobulin; 1:3000 dilution) was added and incubated at room temperature for 1 hour, and bound antibody was visualized using an enhanced chemiluminescence system (Amersham Life Science, Arlington Heights, IL, USA).

### Enzyme-linked immunosorbent assay

The levels of proinflammatory cytokines in the sera or cultured medium were assessed using ELISA kits (eBioscience, San Diego, CA, USA) as described previously^34^. The optical density was determined by an ELISA reader (Molecular Devices, Menlo Park, CA, USA) at 450 nm wavelength, and levels of cytokines were determined by regression analysis against a standard curve.

### Luciferase reporter assay for NF-κB

Luciferase reporter gene assay was performed in a method as we previously reported^11^. RAW 264.7 cells were cotransfected with NF-κB reporter plasmid and renilla plasmid using Lipofectamine2000 DNA transfection reagent following the manufacturer’s protocol (life technologies). 18 h after transfection, the cells were treated in the presence of l% Ti particles with or without addition of 500 ng/ml PGRN, and luciferase activity was measured 24 h later using Dual-Luciferase® Reporter Assay System according to the manufacturer’s instructions (Promega).

### Statistical analysis

Results were expressed as mean values ±S.E.M. Statistics were conducted as Student’s t-test using SPSS software (SPSS Inc, Chicago, IL). P < 0.05 was considered statistically significant.

## Results

### Titanium particles induce PGRN expression both *in vitro* and *in vivo*

To investigate whether Titanium particles (Ti) affect the expression pattern of PGRN, RAW246.7 cells were cultured with and treated by micron-size pure titanium particles (5 × 10^6 ^particles/ml), and the expression of PGRN was measured at various time points. 6 h after Ti stimulation, mRNA levels of PGRN was dramatically increased (Fig. 1B). PGRN protein expression level was also significantly increased following Ti treatment (Fig. 1C). To determine whether Ti affected the expression of PGRN *in vivo*, the mouse air pouch model with or without stimulation of Ti was established and immunohistochemistry staining was performed. As revealed in Fig. 1D, PGRN expression in the Ti-stimulated air pouch membrane was elevated in comparison with the non-Ti-stimulated membrane. Collectively, PGRN expression was enhanced by Ti stimulation both *in vitro* and *in vivo*.

### Deficiency of PGRN leads to an exaggerated inflammatory phenotype in the Ti-induced mouse air pouch model

**Figure 2:**
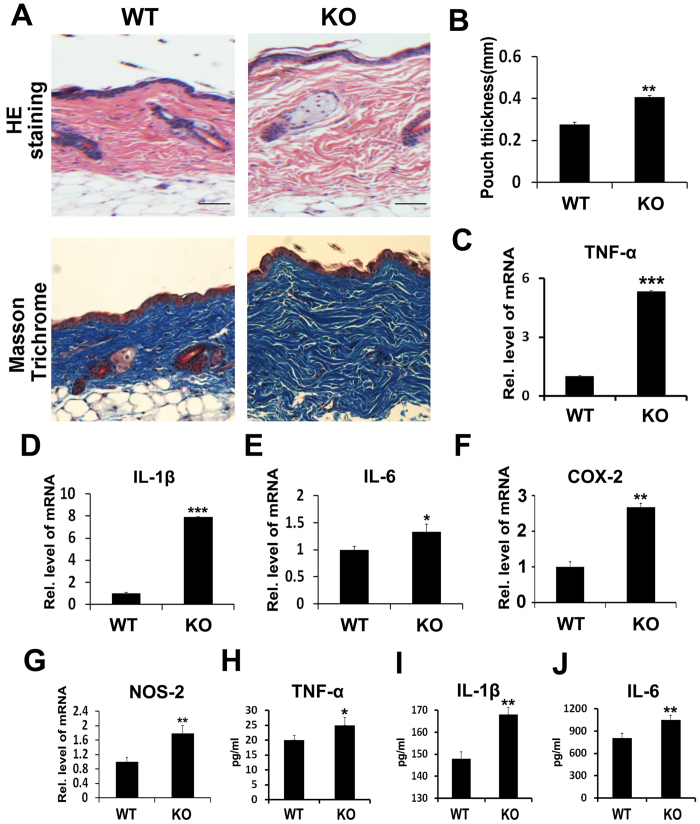
Deficiency of PGRN leads to exaggerated inflammatory phenotype in Ti-induced mouse air pouch model. (**A**) HE staining and Masson Trichrome staining of air pouch membrane. Scar bar = 100 μm. (**B**) Statistic analysis of skin thickness in both wild type mice and PGRN knockout mice according to the HE staining. (**C–G**) mRNA levels of proinflammatory mediators TNFα, IL-1β, IL-6, COX-2 and NOS-2 in Ti-induced inflammatory membranes of wild type mice and PGRN knockout mice, measured by real-time PCR. The units are arbitrary, and the normalized values were calibrated against the wild type control, and each real-time PCR was performed in triplicate. (**H–J**) Serum levels of proinflammatory cytokines TNFα, IL-1β and IL-6 in Ti-induced air pouch model of PGRN knockout mice *vs* wild type mice, assayed by ELISA. Values are the normalized mean ± SEM; *p < 0.05, **p < 0.01 and ***p < 0.001. Six mice were used in each group.

To determine the potential role of endogenous PGRN in Ti-mediated inflammation, the Ti stimulated mouse air pouch model was established in both PGRN knockout (KO) and wild type (WT) mice. As shown in Fig. 2A,B, both H&E and Masson Trichrome staining showed that the air pouch membrane in KO mice was significantly thicker than that of WT mice. To further confirm the difference between KO and WT mice, mRNAs were extracted from the skin tissues and pro-inflammatory molecules, including TNFα, IL-1β, IL-6, COX-2 and NOS-2 were tested by real time PCR assay. As indicated in Fig. 2C–G, the relative mRNA levels of these molecules in the interface membrane of the air pouch were remarkably increased in KO mice compared to WT mice. Additionally, serum levels of TNFα, IL-1β and IL-6 were also significantly higher in KO mice than those in WT mice, assayed by ELISA (Fig. 2H–J). Taken together, loss of PGRN leads to the exaggerated inflammation induced by Ti *in vivo*.

### PGRN suppresses Ti-induced inflammation in an mouse air pouch model

**Figure 3:**
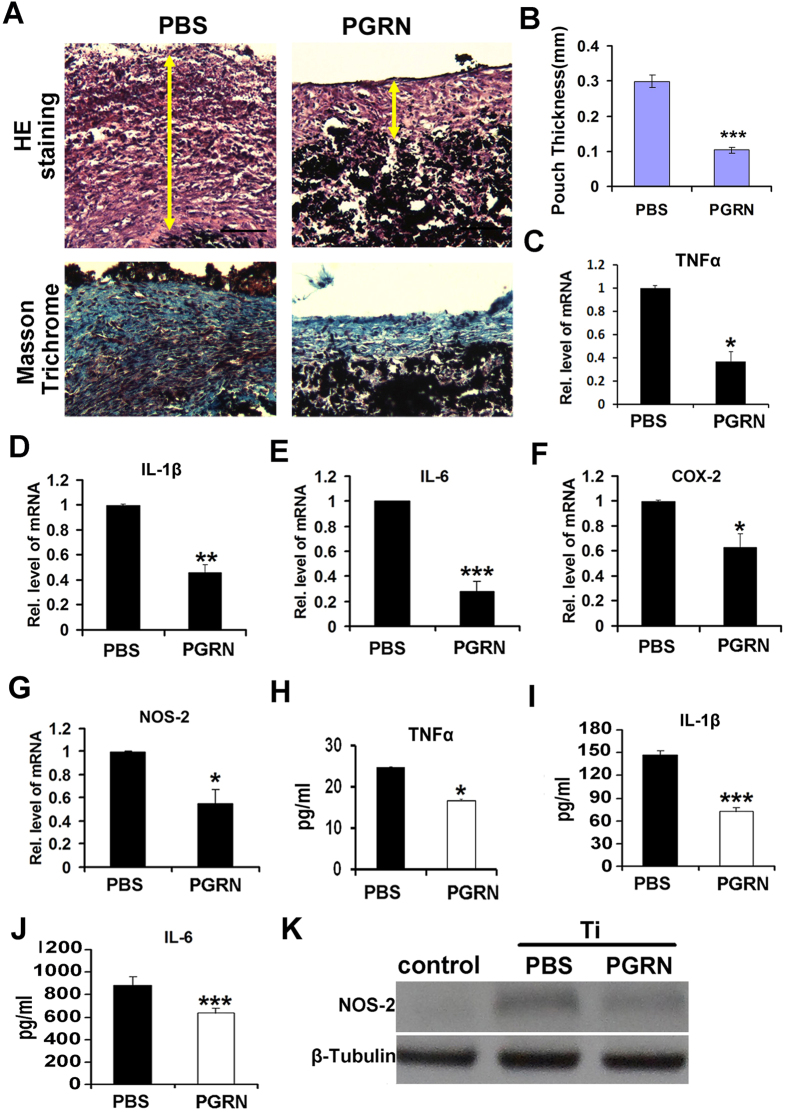
PGRN suppresses Ti-stimulated inflammatory responses in mouse air pouch model. (**A**) HE staining and Masson Trichrome staining of air pouch membrane. Scar bar = 100 μm. (**B**) Statistic analysis of skin thickness in both PBS-treated mice and PGRN-treated mice according to HE staining. (n = 6, **p < 0.01) (**C–G**) mRNA levels of proinflammatory mediators TNFα, IL-1β, IL-6, COX-2 and NOS-2 in PBS- and PGRN treated mice skin, measured by real-time PCR. The units are arbitrary, and the normalized values were calibrated against the PBS control, and each real-time PCR was performed in triplicate. (**H–J**) Serum levels of proinflammatory cytokines TNFα, IL-1β and IL-6 in PBS- and PGRN-treated mice in Ti-stimulated air pouch model, assayed by ELISA. (**K**) Expression of NOS-2 in control, PBS and PGRN treated Ti-induced mice air pouch membrane, detected by Western blotting. Values are the normalized mean ± SEM; *p < 0.05, **p < 0.01 and ***p < 0.001. Six mice were used in each group.

Given that loss of PGRN led to severe inflammation in the Ti-induced mouse air pouch model, we next sought to determine whether recombinante PGRN could prevent the Ti-induced inflammation. We established the air pouch model in WT mice, and injected 0.5 ml PBS or PGRN (200 μg/kg) daily until the mice were sacrificed. As shown in Fig. 3A,B, both H&E and Masson Trichrome staining showed the thickness of the membrane in the PGRN-treated group was significantly reduced compared to the PBS control group. In addition, PGRN significantly reduced the mRNA levels of proinflammatory molecules (Fig. 3C–G). Furthermore, the serum levels of TNFα, IL-1β and IL-6, were also significantly reduced following PGRN administration, assayed by ELISA (Fig. 3H–J). The protein level of NOS-2 was largely diminished by recombinant PGRN, assayed by Western blotting (Fig. 3K). Collectively, these sets of assays clearly demonstrated that recombinant PGRN effectively inhibited Ti-induced inflammation *in vivo*.

### PGRN suppresses Ti-induced inflammatory osteolysis in both *ex vivo* and *in vivo* models

**Figure 4:**
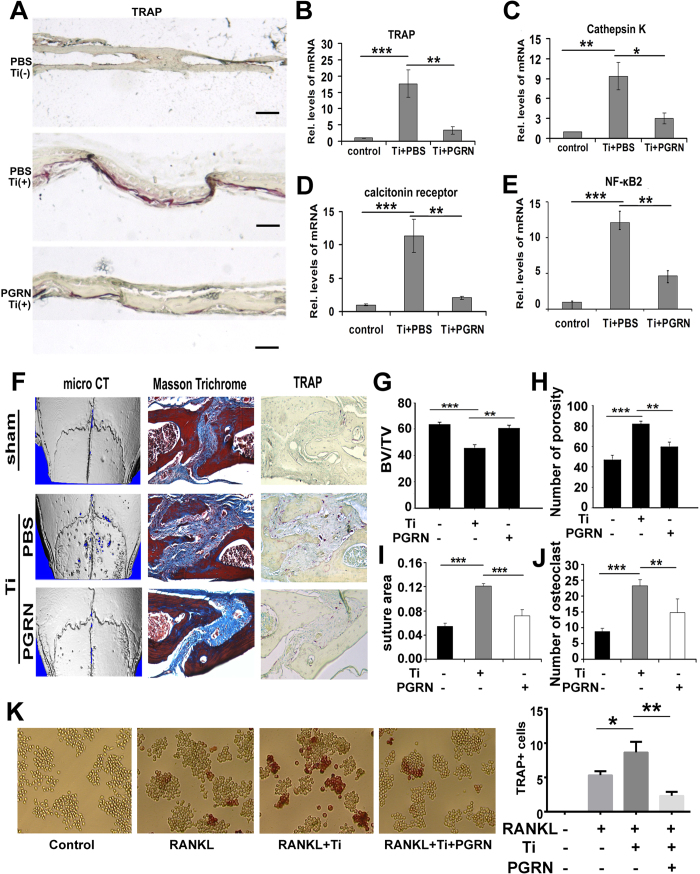
PGRN suppresses Ti-induced inflammatory osteolysis *ex vivo* (**A–E**) and *in vivo* (**F–J**) in mouse calvarial osteolysis model. (**A**) Tartrate-resistant acid phosphatase (TRAP) staining of calvarial bone. TRAP staining was performed at least three sections per group. (**B–D**) mRNA levels of osteoclastogenesis makers: TRAP, Cathepsin K and Calcitonin receptor were extracted from calvarial bone of control group, PBS-treated group and PGRN-treated group, measured by real-time PCR. (**E**) mRNA levels of NF-κB2 from RAW264.7 cells with or without PGRN treatment in presence of Ti, measured by real-time PCR. (**F**) Representative micro-computed tomography (CT) three-dimensional reconstructed images, Masson Trichrome and TRAP staining from each group. (**G–H**) Statistic analysis of BV/TV and number of porosity according to the micro-CT analysis, respectively. (**I**) Sagittal suture area analysis according to the Masson Trichrome staining. (**J**) Statistic analysis of cell number of osteoclast according to the TRAP staining. (**K**) Left panel: representative image of TRAP staining. RAW264.7 cells were cultured under various conditions, as indicated, for 7days to differentiate into osteoclast. TRAP+ cells indicated osteoclast. One cell contains at least 3 nucleus are considered as osteoclast. Right panel: mean number of TRAP-positive cells. Values are mean ± s.d. *p < 0.05, **p < 0.01 and ***p<0.001.

Calvaria from 5-day old WT mice were dissected and cultured in the conditioned medium for 4 days. The conditioned medium was composed of stimulated medium from Ti-activated RAW264.7 cells diluted (1:1) in serum-free DMEM, in the presence or absence of PGRN. The control group of calvaria was cultured in normal DMEM medium. As illustrated in Fig. 4A, TRAP staining was observed as scattered and minimal distribution on bone surface when calvaria were cultured in normal DMEM medium alone (control). However, addition of stimulated medium from Ti-activated RAW264.7 cells caused intense TRAP staining on the bone surface, which extended into adjoining areas. Some regions where TRAP-positive cells localized were pitted, which implied active osteoclastic bone resorption. TRAP staining of calvaria bones was significantly reduced in the PGRN treatment group. Moreover, mRNAs from calvaria of each group were collected and real time PCR was performed for osteoclastic biomarkers including TRAP, Cathepsin K and Calcitonin receptor. As shown in Fig. 4B–D, Ti-activated medium significantly elevated those aforementioned molecules in calvaria, while this elevation was markedly abolished by recombinant PGRN. It is well established that NF-κB2 plays a critical role in the osteoclastogenesis. To further reveal the inhibitory ability of PGRN in osteoclastogenesis, we stimulated RAW264.7 cells in presence or absence of PGRN, Ti was found to markedly induce the expression of NF-κB2 gene , while addition of PGRN largely abolished this induction(Fig. 4E). Taken together, PGRN inhibits Ti mediated osteoclastogenesis *ex vivo*.

To further investigate the protective role of PGRN in Ti-induced osteolysis, a mouse calvaria osteolysis model was established. 200 μg/kg of PGRN or PBS were then injected intraperitoneally every day.calvaria were harvested 8 days after operation. Micro CT showed that Ti exaggerated calvaria osteolysis, and Masson trichrome staining illustrated Ti could cause enlargment of the sagittal suture (Fig. 4F). In addition, TRAP staining also indicated Ti could promote osteoclastogenesis in the calvaria osteolysis model. However, addition of PGRN largely reduced the effect of Ti-induced osteolysis (Fig. 4F). Furthermore, we analyzed the BV/TV and the extent of porosity, respectively. Fig. 4G,H demonstrates that PGRN effectively prevented Ti induced bone loss and porosity, and Fig. 4I,J reveals that Ti-mediated increases in sagittal suture area and TRAP positive cell number were significantly reduced by PGRN. To further determine whether PGRN could inhibit osteoclastogenesis *in vitro*, we cultured RAW264.7 cells in absence(control) or presence of 50 ng/ml RANKL, 50 ng/ml RANKL and 1% titanium particles, 50 ng/ml RANKL and 1% titanium particles and 500 ng/ml PGRN for 7 days, followed by TRAP staining. As demonstrated in Fig. 4K, Ti enhanced RANKL-mediated osteoclast formation, whereasPGRN significantly inhibited Ti-enhanced osteoclast differentiation. Taken together, these results suggest that PGRN may have a therapeutic effect on the Ti-induced inflammatory osteolysis.

### PGRN suppresses Ti-induced inflammation by inhibiting TNF-α signaling *in vitro*

**Figure 5:**
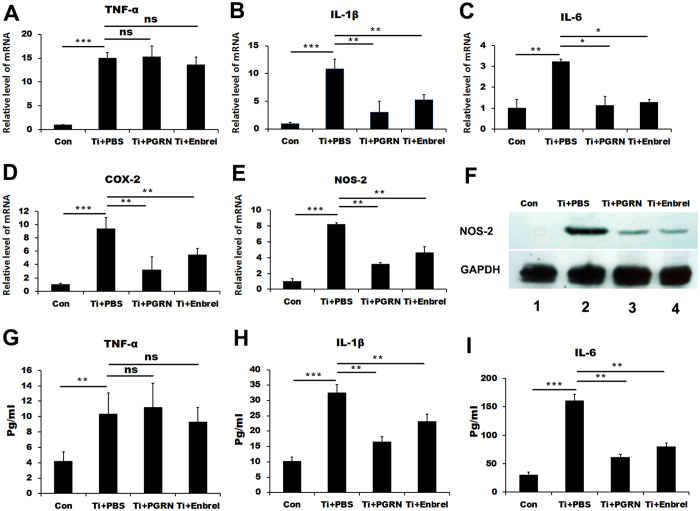
Similar to etanercept, PGRN suppresses Ti-induced expressions of TNFα downstream inflammatory mediators. (**A–E**) mRNA levels of TNFα and its downstreaminflammatory mediators IL-1β, IL-6, COX-2 and NOS-2 from PBS-, PGRN-, or etanercept-treated RAW264.7 cells in presence of Titanium were measured by real-time PCR. (**F**) The levels of NOS-2 in RAW264.7 cells in presence of Ti with or without PGRN or etanercept, assayed by Western blotting. GAPDH was used as internal control. (**G–I**) The levels of TNFα, IL-1β and IL-6 in the medium collected from the RAW264.7 cells cultured in presence of Ti particles with or without PGRN or etanercept assayed by ELISA. **<0.01, NS = No statistic significance.

Since TNF-α plays a predominate role in the Ti-induced inflammatory cascade^35^,^36^,^37^, and PGRN is known to have anti-TNF activity in various conditions, we next determined whether PGRN mediated suppression of Ti-induced inflammation was through blocking TNF-α activity. In this case, we chose etanercept (Enbrel), a clinical TNF-α inhibitor, as positive control. RAW264.7 cells were stimulated by Ti for 6 h in the presence of 500 ng/ml recombinant PGRN or PBS or Enbrel, and the expressions of inflammatory cytokines were then analyzed by real-time PCR. Figure 5A reveals that similar to etanercept TNF-α inhibitor, PGRN did not reduce the gene expression of TNF-α under such conditions. However, both PGRN and etanercept significantly reduced the gene levels of Ti-induced TNF-α downstream inflammatory cytokines, including IL-1β, IL-6, COX-2 and NOS-2 (Fig. 5B–E). In addition, Western blot assay revealed that NOS-2 was almost undetectable, both PGRN and etanercept dramatically decreased the level of Ti-induced NOS-2 (Fig. 5F). Furthermore, both PGRN or etanercept significantly reduced the secretions of Ti-stimulated IL-1β and IL-6 (Fig. 5H–I), although they had no effect on TNFα production. Collectively, these results suggest that PGRN acts as a TNFα inhibitor (i.e. etanercept) and inhibits Ti-induced inflammatory response under this condition.

### PGRN suppresses Ti-induced activation of NF-κB signaling

**Figure 6:**
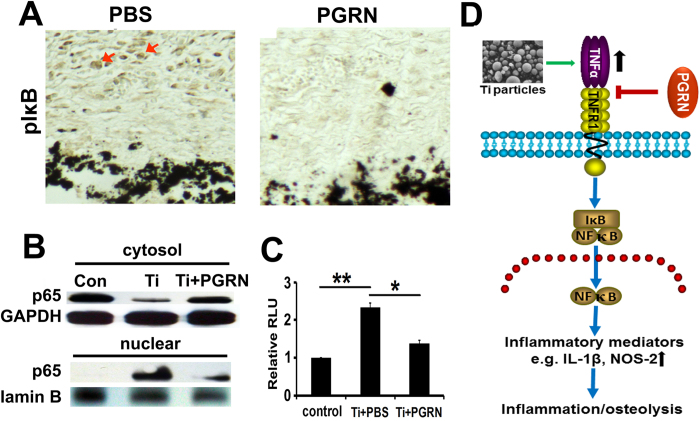
PGRN suppresses Ti-stimulated activation of NF-κB pathway. (**A**) The immunohistochemistry staining of pIκB in Ti-induced air pouch membrane with or without PGRN treatment. Arrows indicate positive staining. (**B**) Nuclear translocation of NF-κB p65 in RAW264.7 cells in response to Ti stimulation and PGRN treatment, assayed by Western blotting. Cytosol and nuclear extracts were extracted from RAW264.7 cells in absence or presence of Ti, with or without PGRN treatment, as indicated. GAPDH and lamin B were used as cytoplasmic and nuclear internal controls, respectively. (**C**) PGRN inhibits Ti-stimulated transactivation of NF-κB specific reporter gene in RAW264.7 cells. RAW264.7 cells were transfected with NF-κB reporter gene and were cultured in the absence or presence of Ti with or without PGRN, as indicated, for 48 hours, and the luciferase activity was measured. Data were presented as fold of changes over the control group after being normalized with renilla activity. *p < 0.05, **p < 0.01. (**D**) Schematic diagram of the mechanism by which PGRN inhibits Ti-induced inflammation and osteolysis. Green and black arrow indicates “stimulation” and “increase”, respectively, whereas red “├” indicates “inhibition”.

Given that TNFα exerts its inflammatory action through activation of NF-κB transcription factor, we next examined whether PGRN inhibited Ti-induced activation of the NF-κB pathway. As shown in Fig. 6A, PGRN treatment markedly reduced the signal of Ti-activated pIκB in the Ti-induced air pouch membrane by immunohistochemistry staining.

To examine whether PGRN affected Ti-induced nuclear translocation of NF-κB transcription factor, RAW264.7 cells were cultured in the presence of Ti with or without recombinant PGRN for 30 minutes. After that, cytosol protein and nuclear protein were extracted separately and Western blot for NF-κB p65 was performed. As shown in Fig. 6B, p65 was predominately expressed in the cytoplasm in the untreated cells, while p65 was dramatically decreased in cytosol and significantly increased in the nucleus following Ti stimulation.

To test whether PGRN could affect NF-κB activity, NF-κB specific luciferase reporter gene was performed. As illustrated in Fig. 6C, Ti could significantly transactivate this reporter gene, whereas Ti-mediated activation of NF-κB reporter gene was significantly inhibited by recombinant PGRN. Collectively, these results indicated that PGRN suppressed Ti-induced activation of NF-κB signaling pathway.

## Discussion

TJA is a common strategy in clinic for therapy of osteoarthritis, rheumatoid arthritis and other joint diseases^38^. However, there are various risk factors which may lead to failure of the artificial joint. Although the underlying mechanisms are not completely known, several factors, including infection, aseptic loosening, low bone mineral density and so on, have been well accepted for failure of the implant in TJA^39^,^40^,^41^,^42^. To date, periprosthetic osteolysis followed by aseptic loosening still remains the major cause of TJA failure and frequently leads to revision surgery^30^,^43^. Previous clinical studies have implied participation of Ti and other wear debris in prosthetic loosening following TJA^44^,^45^. In addition, it was reported that Ti particle could induce TNFα release^46^, and TNFα serves as one of routine parameters in the inflammatory osteolytic process^47^. TNFα has been extensively studied in inflammatory diseases and anti-TNFα agents have been shown to inhibit inflammatory osteolysis^9^,^48^.

PGRN, an antagonist of TNFα, is known to be expressed in various cells and has anti-inflammatory activity in various conditions^12^,^49^,^50^,^51^.In this study, we found that Ti could significantly induce PGRN expression both in RAW264.7 cells and in a mouse air-pouch model of inflammation, which implied that PGRN might also play an anti-inflammatory role in wear debris-mediated pathological processes. Indeed, deletion of PGRN significantly enhanced the Ti-induced inflammatory response, as indicated by the phenotype and elevated levels of pro-inflammatory cytokines, such as TNFα, IL-1β and IL-6, both at the mRNA expression and protein synthesis levels. In addition, recombinant PGRN effectively prevented Ti-induced inflammation. NOS-2 level was elevated within the interface membrane obtained from aseptically loose prostheses^47^. Moreover, suppression of NOS-2 indicated the alleviation of wear debris-induced inflammation^52^,^53^,^54^. In the present study, we also found that NOS-2 levels were enhanced by Ti, a finding which is consistent with previous publications, and this Ti-induced NOS-2 expression was markedly inhibited by PGRN.

To better understand the role of PGRN in the inflammatory osteolysis, we cultured neonatal mice calvariae in Ti-stimulated medium, and found that osteoclastogenesis mediated by the medium was largely blocked by PGRN, which implied that PGRN could suppress the activity of cytokines in the medium, or PGRN could directly suppress osteoclastogenesis induced by inflammatory cytokines. These results were further validated in the *in vivo* calvarial osteolysis mouse model. These findings are also consistent with the reports that PGRN and PGRN-derived Atsttrin inhibited osteoclastogenesis and protected bone erosion in inflammatory arthritis^13^,^14^,^55^.

Although PGRN significantly reduced the levels of Ti-induced inflammatory cytokines in a mouse air pouch model, including TNFα (Fig. 3), we were surprised to find that both the mRNA and protein levels of TNFα remained unchanged after addition of PGRN in RAW264.7 cells (Fig. 6). However, the downstream molecules of TNFα signaling, such as IL-1β, IL-6, COX-2 and NOS-2, were largely inhibited by PGRN both *in vivo* and *in vitro*, suggesting that PGRN inhibited Ti-induced inflammation through inhibiting TNFα activity in RAW264.7 cells and inhibited Ti-induced inflammatory osteolysis through inhibiting both TNFα activity and production *in vivo*. As expected, both *in vivo* and *in vitro* data indicated that PGRN could significantly inhibit Ti-induced activation of NF-κB pathways, including the phosphorylation of I κB, nuclear translocation of NF-κB p65, and the NF-κB specific reporter gene assays.

PGRN binds to several members of the TNFR family, including TNFR1 and TNFR2^56^,^57^,^58^,^59^,^60^. TNFR1 and TNFR2 do not share homology in the cytoplasmic domains but exhibit a low degree of similarity in the ligand-binding region located in the extracellular domains, which suggests that they are capable of inducing distinct cellular responses^61^. PGRN exhibited approximately 600-fold higher binding affinity to TNFR2 than TNFα^14^,^62^. In line with these findings, PGRN-mediated signaling and protective roles were reported to primarily depend on TNFR2 pathway in various kinds of diseases models and conditions, including osteoarthritis^11^, inflammatory bowel diseases^12^, fracture healing^25^ and endoplasmic reticulum (ER) stress-mediated apoptosis^60^ Interestingly, a recent report demonstrated that PGRN-mediated hepatic insulin resistance through TNFR1 via activating NF-κB signaling^63^. In this study, we found that PGRN, similar to etanercept, effectively inhibited the Ti-induced inflammatory response (Fig. 6). Additionally, blocking TNFR2 signaling with its specific antibody did not affect PGRN inhibition of Ti-induced expression of NOS-2 (data not shown). These results indicate that PGRN inhibition of Ti-induced inflammation probably occurs through the TNFR1 pathway. In addition, PGRN inhibited TNFα-mediated activation of its canonical NF-κB signaling in the Ti-induced inflammatory response. It appears that PGRN binds to TNFR1, followed by the activation or inhibition of NF-κB pathways, which strictly depends on the microenvironment of the specific disease. It is postulated that PGRN acts as an inhibitor of TNFα via competing with TNFα for binding TNFR1, and inhibits TNFα-mediated activation of NF-κB, under the conditions in which TNFα plays a dominant role, such as Ti-induced inflammatory osteolysis (this study). In contrast, PGRN (especially its degradative fragments) may also directly bind to TNFR1 and activate the NF-κB pathway under other conditions, such as diabetes^63^.

Based on the findings in this study, as well as previous reports, we propose a model for summarizing the protective role of PGRN and its regulation in Ti particle-induced inflammatory osteolysis (Fig. 6D): Ti particles lead to the release of TNFα, a master regulator of proinflammatory cytokine cascades, that activates classical TNFR1/NF-κB pathways and in turn the release of downstream inflammatory mediators, such as IL-1β and NOS-2. PGRN prevents Ti-induced inflammatory osteolysis by at least partial inhibition of the binding of TNFα to TNFR1. Collectively, the findings reported in this study not only provide new insight into the molecular mechanisms underlying Ti-induced inflammatory osteolysis, but may also present PGRN or its derivatives as a new therapeutic strategy for the prevention of aseptic loosening.

## Additional Information

**How to cite this article**: Zhao, Y.-p. *et al*. Progranulin suppresses titanium particle induced inflammatory osteolysis by targeting TNF α signaling. *Sci. Rep*. **6**, 20909; doi: 10.1038/srep20909 (2016).
